# The Crystal Structure of Monovalent Streptavidin

**DOI:** 10.1038/srep35915

**Published:** 2016-12-21

**Authors:** Min Zhang, Sangita Biswas, Wenbin Deng, Hongjun Yu

**Affiliations:** 1 Medical College, Hubei University of Arts and Science, Xiangyang, Hubei, China; 2 Department of Biochemistry and Molecular Medicine, School of Medicine, University of California, Davis, CA, USA; 3 Department of Biology, Brookhaven National Lab, NY, USA

## Abstract

The strong interaction between streptavidin (SA) and biotin is widely utilized in biotechnological applications. A SA variant, monovalent SA, was developed with a single and high affinity biotin-binding site within the intact tetramer. However, its structural characterization remains undetermined. Here, we seek to determine the crystal structure of monovalent SA at 1.7-Å resolution. We show that, in contrast to its ‘close-state’ in the only wild-type subunit, the L3,4 loops of three Dead SA subunits are free from crystal packing and remain in an ‘open state’, stabilized by a consistent H-bonding network involving S52. This H-bonding network also applies to the previously reported open state of the wild-type apo-SA. These results suggest that specific substitutions (N23A/S27D/S45A) at biotin-binding sites stabilize the open state of SA L3,4 loop, thereby further reducing biotin-binding affinity. The general features of the ‘open state’ SA among different SA variants may facilitate its rational design. The structural information of monovalent SA will be valuable for its applications across a wide range of biotechnological areas.

Streptavidin (SA) is a tetrameric protein derived from the bacterium *Streptomyces Avidini*, which exhibits extraordinary affinity for biotin^1^. The streptavidin-biotin system is acknowledged as one of the most stable noncovalent interactions and has wide applications in the field of biotechnology such as molecular-labeling, molecular- localization and targeted drug delivery^3^,^4^.

Recognizing the utility of this system in different applications, extensive engineering efforts have been made to modify the SA system. For example, to reduce the immunogenicity of SA in clinical use, a low immunogenic SA mutant has been designed and structurally investigated^5^,^6^. Similarly, despite the high affinity of biotin-streptavidin, biotin dissociation results from low endosomal pH^7^ or being attached to nanoparticles^8^. This issue was overcome by traptavidin, a SA variant bearing S52G/R53D mutants^9^, which was also explained later by its crystal structural^10^. Moreover, four biotin-binding sites of SA tetramer can multimerize biotinylated ligands, affecting their function or causing target aggregation^11^. Howarth *et al*.^12^ reported the production of monovalent streptavidin with N23A/S27D/S45A triple mutants in three Dead subunits. This chimeric SA variant has a single biotin-binding site with high (femtomolar) affinity and is still in tetramer form. However, the information regarding the structural basis of its function is still missing.

In this study, we report the 1.65 Å crystal structure of monovalent SA with three Dead subunits containing N23A/S27D/S45A triple mutants. In this structure, the L3,4 loops of three Dead subunits, all adopted open conformation in contrast to the close conformation of the only wild type subunit in the same SA tetramer. A consistent H-bonding network was found to stabilize the open L3,4 among three Dead subunits, which involves S52 on the L3,4 loop. This H-bonding network also applies to the apo-SA with open L3,4 and may be a general feature of the open conformation of SA. These results suggest that the substitutions at biotin-binding sites (N23A/S27D/S45A) may stabilize the open conformation of the L3,4 loop, and further reduce biotin-binding. These insights from monovalent SA structure promote our understanding of this widely used biotechnological system and can facilitate its rational modification and application.

## Results

### Overview of monovalent streptavidin structure

**Figure 1:**
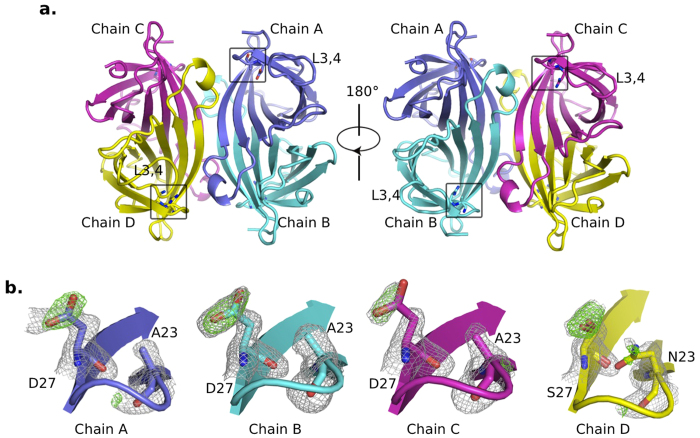
Structure of monovalent streptavidin. (**a)** Structure of the monovalent streptavidin, with the three Dead subunits (chain A, B and C) represented by in blue, cyan and magenta color respectively, and the WT subunit (chain D) in yellow. (**b**) Unbiased electron densities at mutation sites 23 and 27 were generated with their side chains deleted before molecular replacement and determining their identities. Grey mesh, *2fo-fc* map contoured at 1.1 sigma; green mesh, *fo-fc* map contoured at 3 sigma.

Monovalent streptavidin structure was determined at 1.65 Å by molecular replacement using wild-type SA tetramer (PDB id: 3RY1) as the search model. It was with P1 space group (Table 1), containing a tetramer in the asymmetric unit. Each subunit is composed of an eight-stranded antiparallel β-barrel (Fig. 1a), typical of SA structures previously reported^13^. To identify the only wild-type SA subunit (WT subunit) from the other three inactive subunits (Dead subunits with N23A/S27D/S45A triple mutations), the side chains at residue 23 and 27 of wild-type SA (PDB id: 3RY1) were removed and used for molecular replacement. After solving the phase, an unbiased map at reside 23 and 27 was subsequently generated to identify the missing side chain (Methods and Fig. 1b). By this analysis, the chain A, B and C were identified as Dead subunits and chain D was identified as wild-type one (WT subunit). Notably, density around residue 45 was not included for this analysis because it locates to the L3,4 loop, which exhibited variable conformations.

### Different conformations of L3,4 loop between wild type and Dead subunits

**Figure 2:**
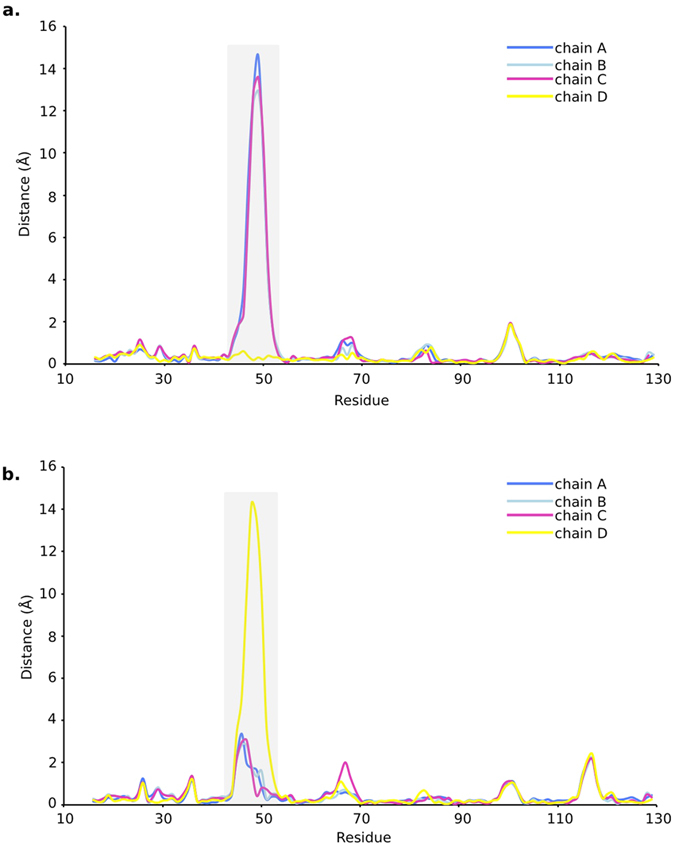
Cα deviations after structural alignment of the four subunits (chain A–D in this study) with two previously reported conformations of SA. (**a**) Comparison with biotin-close conformation (chain A of pdb id: 1MK5). (**b**) Comparison with apo-open conformation (chain D of pdb id: 3RY1). The curves are colored identical to the subunit colors in Fig. 1.

**Figure 3:**
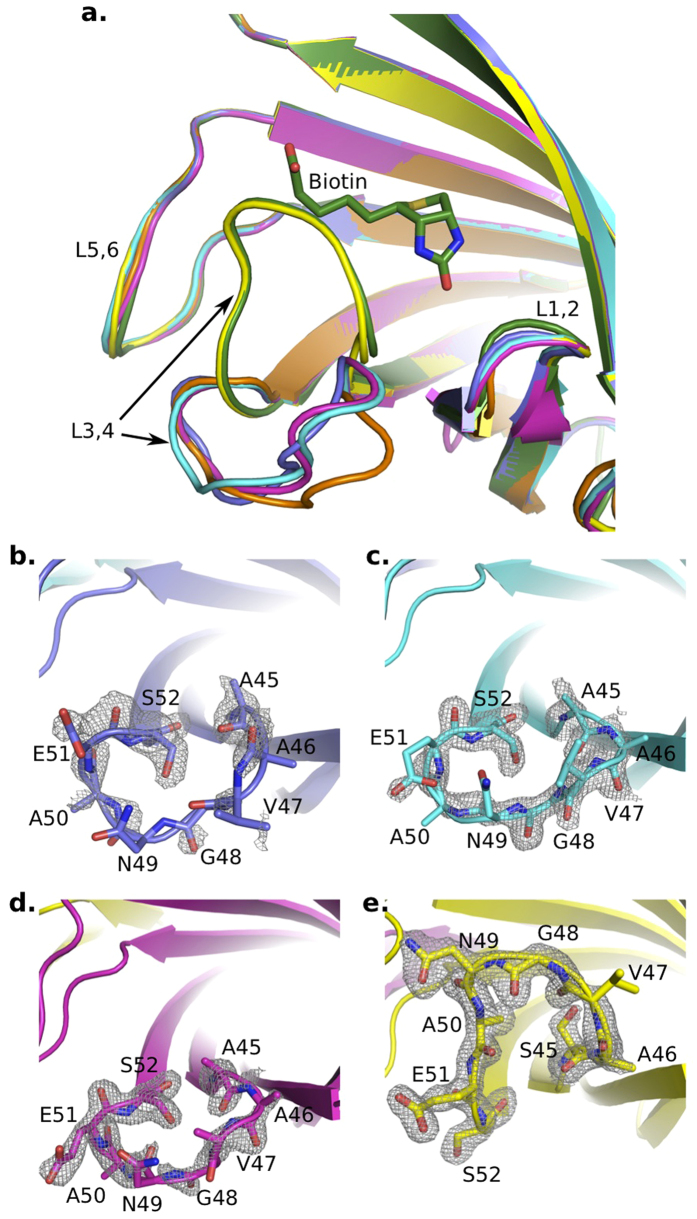
Different L3,4 conformations between Dead and WT SA subunits. (**a**) Four monovalent SA subunits (chain A–D, colored identical to that seen in Fig. 1) were superposed with two previously known SA states: biotin-close conformation (dark green, chain A of pdb id: 1MK5), and the apo-open conformation (orange, chain D of pdb id: 3RY1). (**b–e)** electron densities (grey mesh, *2fo-fc* map contoured at 1.1 sigma) around the L3,4 of four monovalent SA subunits.

The biotin binding sites are located at one end of these β-barrels (Fig. 1a). L3,4 (residues 45–52) the loop connecting β-strands 3 and 4, exhibited different conformations among four subunits (Fig. 1a). The L3,4 loop is usually flexible in apo-SA, adopting either ‘open’ or ‘close’ conformation while it always folds over bound biotin (in ‘close’ conformation)^10^,^13^. To investigate the different conformations of L3,4 in monovalent SA, we then compared its four subunits with apo-SA open conformation (chain D of pdb id: 3RY1, referred as ‘apo-open conformation’ in later description), and biotin-bound ‘close’ conformation (chain A of pdb id: 1MK5, referred as ‘biotin-close conformation’ in later description). This revealed that WT subunit (chain D) resembles the biotin-close conformation while three Dead subunits (chain A, B and C) are comparable to the apo-open conformation (Fig. 2 and Supplementary Table 1). The conformation of the L3,4 loop made the main difference between these two forms. This loop bends over the biotin site in the WT subunit (chain D). However, in all three Dead subunits (chain A, B and C), the L3,4 loops remain open, exposing their ligand binding sites (Fig. 3a). Calculation of electron density near this loop region suggested the reliable tracing of main chain in four subunits, though the density was relatively weak in chain A (Fig. 3b).

### The ‘open’ L3,4 loops are consistently stabilized in all Dead streptavidin subunits

**Figure 4:**
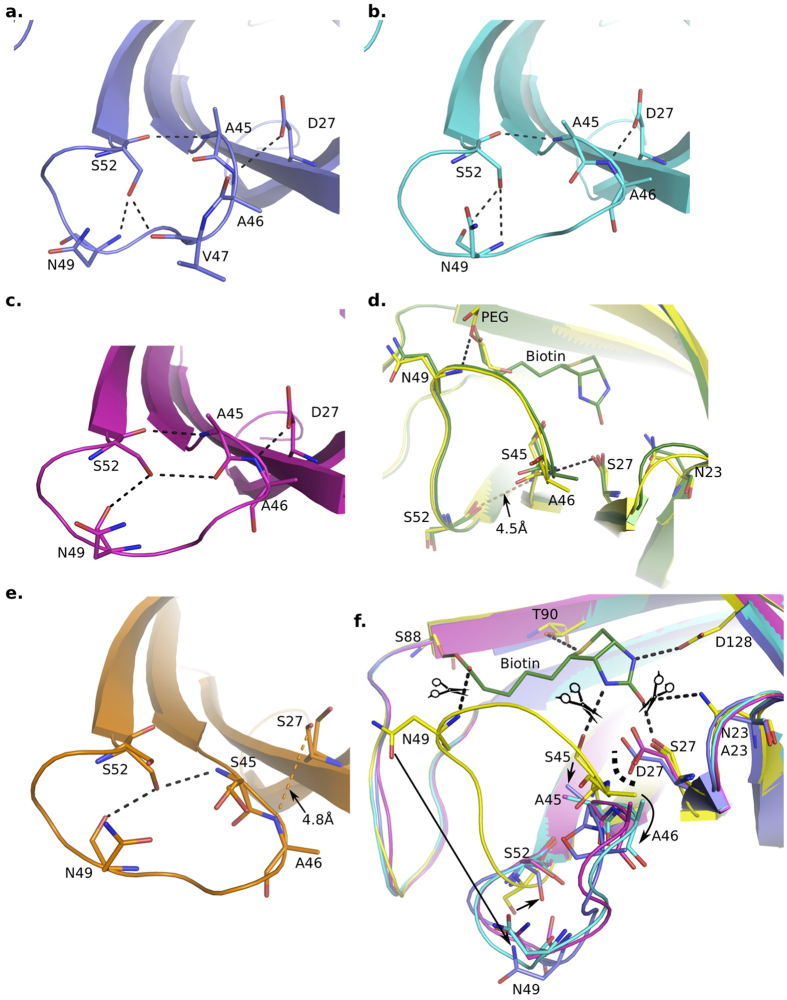
Effects of N23A/S27D/S45 mutations. Different subunits are colored identical to that seen in Fig. 3a. **(a–c)** A universal H-bonding network to stabilize the open L3,4 of Dead subunits with N23A/S27D/S45 triple mutants. H-bonds are indicated by dashed lines. (**d**) Comparison between WT subunit (yellow) and biotin-close conformation (dark green, chain A of pdb id: 1MK5). A PEG molecule was identified in the ligand-binding site of WT subunit, which involves an H-bond with N49 main chain (black dashed lines). The H-bonds observed in Dead subunits (panel **a–c**) can’t be maintained due to conformational change of L3,4. (**e**) H-bonding network (black dashed lines) observed in apo-open conformation (chain D of pdb id: 3RY1) is comparable to that in Dead subunits (panel **a–c**). (**f**) Comparison of the L3,4 loop and biotin-binding pocket among the 4 subunit structures (panel (**a–d**) in monovalent streptavidin identified the effects of N23A/S27D/S45A in Dead subunits. N23A/S27D/S45A can directly damage the interaction with biotin while S27D mutation may cause shift of L3,4 away from biotin-site into a stabilized open state, further reducing biotin interaction otherwise provided by close L3,4 loop. Curve dash line indicates the effects of S27D mutation. The change of L3,4 loop from open to close conformation is indicated by four arrows. Biotin (dark green stick) was modeled as shown in panel (**d**).

Loops between streptavidin β-strands are often involved in crystal packing^13^. We therefore evaluated the crystal packing around the L3,4 loop in monovalent SA structure. As a result, for the only WT subunit (chain D), there is only one H-bond between S52 main chain at the base of L3,4, and T66 main chain from L4,5 loop of neighboring subunit, which may favor the ‘close’ conformation of its L3,4 over ‘open’ conformation (Supplementary Fig. 1d and e). However, L3,4 loops within three Dead subunits (chain A, B and C) are free from crystal packing (Supplementary Fig. 1a-c), which enabled us to evaluate direct effects of mutations on L3,4. As we discussed above, L3,4 of all three subunits are consistently open (Fig. 3a). Furthermore, we observed an universal H-bonding network is responsible for stabilizing this open L3,4 conformation in all three Dead subunits: S27D side chain forms a H-bond with main chain of A46; S52 side chain interacts with main chains of N49 (chain A, B and C) and V47 (chain A only) (Fig. 4a–c); S52 main chain form a H-bond with S45A main chain. Notably, in previously reported wild-type apo-open conformation, we found similar H-bonding interactions within open L3,4, that is, S52 side chain forms H-bonds with N49 main chain and S45 main chain (Fig. 4e). In contrast, in the close L3,4 observed in WT subunit (chain D), these interactions cannot be maintained because S52 rotates almost 180° (Fig. 4d). These results in different SA variants indicated the important role of S52 in stabilizing the open conformation of L3,4.

In the WT subunit, one PEG molecule take up the space which biotin tail occupies and forms a H-bond with the N49 main chain, similar to biotin binding (Fig. 4d). Therefore, the biotin-binding site of WT subunit closely resembles that observed in the biotin-bound state. Further superimposition of these different subunits revealed that S27D mutation may generate steric hindrance with A46 in close L3,4 (Fig. 4f), suggesting this may result in the shift of this loop and form a preferred open conformation maintained by S52.

## Discussion

By solving the structure of monovalent streptavidin, we found substantial conformational changes between the single wild-type subunit and three Dead subunits, localized to their L3,4 loops. Specifically, L3,4 loop folds over the biotin-binding site in WT subunit while it remains in ‘open’ conformation among three Dead subunits (Fig. 3a). The L3,4 loops free from crystal packing (Supplementary Fig. 1) enabled us to evaluate the effects of N23A/S27D/S45A mutations on biotin binding (Fig. 4). We found that S27D may sterically force the L3,4 to shift away from biotin-binding site and stabilize its open conformation through an H-bonding network involving S52 (Fig. 4f). It was proposed that N23A/S27D/S45A directly attenuated the binding of biotin^12^,^14^. Here our analysis suggest the stabilized open L3,4 caused by these mutants may further reduce the binding of biotin, because only close L3,4 can provide extra sites for biotin-binding (Fig. 4f). Moreover, these stabilized open L3,4 loops may also expose the biotin binding site, resulting in fast release of biotin. Consistently, the open L3,4 was previously proposed to explain the fast dissociation of ALiS from SA^15^. All these effects together may account for the substantial reduction of biotin binding to N23A/S27D/S45A mutants^12^. Our results also suggested the important role of S52 in stabilizing the open conformation of L3,4, the ‘lid’ of the biotin-binding pocket. This involves H-bonds between S52, N49 and S45 (or A45 if mutated), which was observed in all three Dead subunits in this study, and also in apo-open wild-type SA (Fig. 4). Previous studies indicated that SA S52G caused reduction of on-rate and off-rate binding constants for ligand binding, and SA structures having S52G mutants all exhibited closed L3,4 lid, regardless of the presence or absence of ligand^10^,^16^. Based on our analysis, these effects of S52G are probably caused by the collapse of the H-bonding network within the open L3,4 and the subsequent destabilization of this open conformation. These insights emphasized the H-bonds within the open L3,4 loop in biotin-binding kinetics, which can be potential target for modulating SA properties to overcome its disadvantages such as non-favored ligand dissociation^7^,^8^.

## Materials and Methods

### Monovalent streptavidin expression and purification

The monovalent streptavidin in tetrameric form was generated as previously described^12^. Briefly, wild-type SA (with His8-tag) and Dead (N23A/S27D/S45A) SA (without His8-tag) were expressed in *E. coli* BL21 (DE3) cells as inclusion bodies. The collected cells were lysed by sonication and the inclusion bodies were isolated and washed twice with washing buffer (20 mM Tris–HCl, pH 8.0, 0.3 M NaCl, 2 M urea) to reach over 80% purity. The purified inclusion bodies pellet of wild-type and mutant streptavidin were both resuspended in solubilization buffer (20 mM Tris–HCl, pH 8.0, 0.3 M NaCl, 8 M urea) and then were centrifuged at 15,000 rpm for 30 min to remove insoluble material. After determining the concentration by OD280 (NanoDrop 2000, Thermo Scientific), the solubilized inclusion bodies of wild-type and mutated streptavidin were mixed in 1:3 ratio. The mixture was then fast diluted (drop by drop) into PBS buffer (10 mM Na_2_HPO_4_, 1.8 mM KH_2_PO_4_, 137 mM NaCl) for the refolding of streptavidin tetramer. Ammonium sulfated gradient precipitation was performed to remove the partially folded streptavidin and some contaminant protein^12^,^17^. Different forms of the SA tetramer were separated through a step gradient elution on Nickel charged-nitrilotriacetic acid (Ni-NTA, Qiagen) affinity chromatography and monovalent streptavidin was eluted in buffer with 100 mM imidazole as previously described^18^. The sample was then incubated with the Proteinase K at the weight ration of 100:1 for 5hr at 20 °C to remove the only his-tag on wild-type SA subunit^18^. The protease-treated sample was further purified through size exclusion chromatography (Superdex 75 column) with Tris-sodium buffer (20 mM Tris-HCl, pH 8.0, 150 mM NaCl) to remove the protease and to achieve more homogeneous state. The purified, tag-free monovalent SA tetramer was concentrated to 10–15 mg/ml and was frozen and stored at −80 °C.

### Crystallization of monovalent streptavidin protein

Crystal screens were set up using commercial screens (Hampton Research) by the hanging drop vapor diffusion methods in 96-well plates at 20 °C, where the drops are mixtures of 0.5 ul of well solution and 0.5 ul protein sample (10 mg/ml, Tris-sodium buffer). Crystals were obtained against a reservoir solution containing 0.1 M Tris pH 8.3, 0.25 M MgCl_2_ and 32% poly ethylene glycol (PEG) 4K. Crystals were then picked and flash-frozen in liquid nitrogen. The diffraction data was collected at BL17U beamline at Shanghai Synchrotron Radiation Facility (SSRF).

### Structural determination

Diffraction data was processed and scaled using Mosflm^19^. To solve the phase and to identify the only wild-type subunit, we used apo-SA tetramer (PDB id: 3RY1) as the search model with all the side chains at mutated sites 23, 27 being deleted. The molecular replacement was performed with MOLREP^20^ (a program for automated molecular replacement where a homologous structure has already been identified) followed by one round of automatic refinement with REFMAC^21^ in CCP4^19^. The *fo-fc* and *2fo-fc* maps at this stage were then carefully analyzed to assign the wild-type and Dead subunits and to build the missing side chains. After that, several round of refinement with REFMAC (a program designed for REFinement of MACromolecular structures) and model adjustment with COOT^22^ (Crystallographic Object-Oriented Toolkit) was performed. Waters were added to the models at the last stage. The statistics for data collection and refinement were summarized in Table 1. Structural figures were made using a molecular graphics and modelling package (PyMOL)^23^.

## Additional Information

**Accession codes:** The coordinate and structure factor have been deposited in the Protein Data Bank under accession code 5TO2. 

**How to cite this article**: Zhang, M. *et al*. The Crystal Structure of Monovalent Streptavidin. *Sci. Rep.* **6**, 35915; doi: 10.1038/srep35915 (2016).

**Publisher's note:** Springer Nature remains neutral with regard to jurisdictional claims in published maps and institutional affiliations.

## Supplementary Material

Supplementary Information

## Figures and Tables

**Table 1:** Data collection and structure refinement statistics of monovalent streptavidin.

	Monovalent SA
**Data collection**	
Space group	*P*1
Cell dimensions	
*a, b, c* (Å)	44.36, 56.74, 57.43
α, β, γ (°)	87.54, 85.89, 67.99
Resolution (Å)	50-1.65 (1.74–1.65)
*R*_sym_ or *R*_merge_ (%)	6.8 (45.8)
*I*/σ*I*	9.8 (2.5)
Completeness (%)	94.8 (93.6)
Redundancy	3.3 (3.3)
**Refinement**	
Resolution (Å)	50.0–1.65
No. reflections	62294
*R*_work_/*R*_free_	20.0/23.3
No. atoms	3907
Protein	3618
Water	282
PEG	7
*B*-factors	25.7
Protein	24.9
Water	35.5
PEG	49.5
R.m.s. deviations	
Bond lengths (Å)	0.010
Bond angles (°)	1.42
Ramachandran plot (%)	
Preferred	96.1
Allowed	3.7
Outlier	0.2

## References

[b1] Laitinen O. H., Hytonen V. P., Nordlund H. R. & Kulomaa M. S. Genetically engineered avidins and streptavidins. Cell. Mol. Life Sci. 63, 2992–3017 (2006). 17086379 10.1007/s00018-006-6288-z PMC11136427

[b2] Green N. M. Avidin and streptavidin. Meth. Enzymol. 184, 51–67 (1990). 2388586 10.1016/0076-6879(90)84259-j

[b3] Laitinen O. H., Nordlund H. R., Hytonen V. P. & Kulomaa M. S. Brave new (strept)avidins in biotechnology. Trends Biotechnol. 25, 269–277 (2007). 17433846 10.1016/j.tibtech.2007.04.001

[b4] Wilchek M., Bayer E. A. & Livnah O. Essentials of biorecognition: The (strept)avidin–biotin system as a model for protein–protein and protein–ligand interaction. Immunol. Lett. 103, 27–32 (2006). 16325268 10.1016/j.imlet.2005.10.022

[b5] Yumura K. . Mutations for decreasing the immunogenicity and maintaining the function of core streptavidin. Protein Sci. 22, 213–221 (2013). 23225702 10.1002/pro.2203 PMC3588917

[b6] Kawato T. . Crystal structure of streptavidin mutant with low immunogenicity. J. Biosci. Bioeng. 119, 642–647 (2015). 25434833 10.1016/j.jbiosc.2014.10.025

[b7] Bruneau E., Sutter D., Hume R. I. & Akaaboune M. Identification of nicotinic acetylcholine receptor recycling and its role in maintaining receptor density at the neuromuscular junction *in vivo*. J. Neurosci. 25, 9949–9959 (2005). 16251443 10.1523/JNEUROSCI.3169-05.2005 PMC6725561

[b8] Swift J. L., Heuff R. & Cramb D. T. A two-photon excitation fluorescence cross-correlation assay for a model ligand-receptor binding system using quantum dots. Biophys. J. 90, 1396–1410 (2006). 16299079 10.1529/biophysj.105.069526 PMC1367290

[b9] Chivers C. E. . A streptavidin variant with slower biotin dissociation and increased mechanostability. Nat. Methods 7, 391–393 (2010). 20383133 10.1038/nmeth.1450 PMC2862113

[b10] Chivers C. E., Koner A. L., Lowe E. D. & Howarth M. How the biotin-streptavidin interaction was made even stronger: investigation via crystallography and a chimaeric tetramer. Biochem. J. 435, 55–63 (2011). 21241253 10.1042/BJ20101593 PMC3062853

[b11] Dundas C. M., Demonte D. & Park S. Streptavidin-biotin technology: improvements and innovations in chemical and biological applications. Appl. Microbiol. Biotechnol. 97, 9343–9353 (2013). 24057405 10.1007/s00253-013-5232-z

[b12] Howarth M. . A monovalent streptavidin with a single femtomolar biotin binding site. Nat. Methods 3, 267–273 (2006). 16554831 10.1038/NMETHXXX PMC2576293

[b13] Le Trong I. . Streptavidin and its biotin complex at atomic resolution. Acta Crystallogr. D Biol. Crystallogr. 67, 813–821 (2011). 21904034 10.1107/S0907444911027806 PMC3169315

[b14] Fairhead M., Krndija D., Lowe E. D. & Howarth M. Plug-and-play pairing via defined divalent streptavidins. J. Mol. Biol. 426, 199–214 (2014). 24056174 10.1016/j.jmb.2013.09.016 PMC4047826

[b15] Terai T. . Artificial Ligands of Streptavidin (ALiS): Discovery, Characterization, and Application for Reversible Control of Intracellular Protein Transport. J. Am. Chem. Soc. 137, 10464–10467 (2015). 26261872 10.1021/jacs.5b05672

[b16] Magalhaes M. L. . Evolved streptavidin mutants reveal key role of loop residue in high-affinity binding. Protein Sci. 20, 1145–1154 (2011). 21520321 10.1002/pro.642 PMC3149188

[b17] Schmidt T. G. & Skerra A. One-step affinity purification of bacterially produced proteins by means of the “Strep tag” and immobilized recombinant core streptavidin. J. Chromatogr. A 676, 337–345 (1994). 7921186 10.1016/0021-9673(94)80434-6

[b18] Zhang M., Shao J., Xiao J., Deng W. & Yu H. A novel approach to make homogeneous protease-stable monovalent streptavidin. Biochem. Biophys. Res. Commun. 463, 1059–1063 (2015). 26074145 10.1016/j.bbrc.2015.06.058 PMC4498676

[b19] Winn M. D. . Overview of the CCP4 suite and current developments. Acta Crystallogr. D Biol. Crystallogr. 67, 235–242 (2011). 21460441 10.1107/S0907444910045749 PMC3069738

[b20] Vagin A. & Teplyakov A. Molecular replacement with MOLREP. Acta Crystallogr. D Biol. Crystallogr. 66, 22–25 (2010). 20057045 10.1107/S0907444909042589

[b21] Pannu N. S., Murshudov G. N., Dodson E. J. & Read R. J. Incorporation of prior phase information strengthens maximum-likelihood structure refinement. Acta Crystallogr. D Biol. Crystallogr. 54, 1285–1294 (1998). 10089505 10.1107/s0907444998004119

[b22] Emsley P., Lohkamp B., Scott W. G. & Cowtan K. Features and development of Coot. Acta Crystallogr. D Biol. Crystallogr. 66, 486–501 (2010). 20383002 10.1107/S0907444910007493 PMC2852313

[b23] Schrodinger L. L. C. The PyMOL Molecular Graphics System, Version 1.3r1. (2010).

