# Supplementary material for: The Crystal Structure of Monovalent Streptavidin
Source: Sci Rep. 2016 Dec 21;6:35915. doi: 10.1038/srep35915 (PMC5175265; doi:10.1038/srep35915)
Supplement: Supplementary Information [file srep35915-s1.pdf]

The Crystal Structure of Monovalent Streptavidin

Min Zhang [a, b], Sangita Biswas [b], Wenbin Deng [b *], Hongjun Yu [c *]

[a] Medical College, Hubei University of Arts and Science, Xiangyang, Hubei, China; [b] Department of Biochemistry and Molecular Medicine, School of Medicine, University of California, Davis, CA, USA; [c] Department of Biology, Brookhaven National Lab, NY, USA.

* Corresponding authors:

wbdeng@ucdavis.edu (W. D.) or yuhongjun05@gmail.com (H. Y.)

Supplementary Figure 1. Crystal packing around four subunits of monovalent streptavidin. Subunits are colored as that in figure 1. L3,4 loops are marked by circles. L3,4 loops in all three Dead subunits are free from packing (**panel a-c**) while for the WT subunit (panel **d, e**), the base (S52) of its L3,4 forms a H-bond with T66 from neighboring chain A. **e**, Zoomed view of the region highlighted by rectangle in panel **d**.

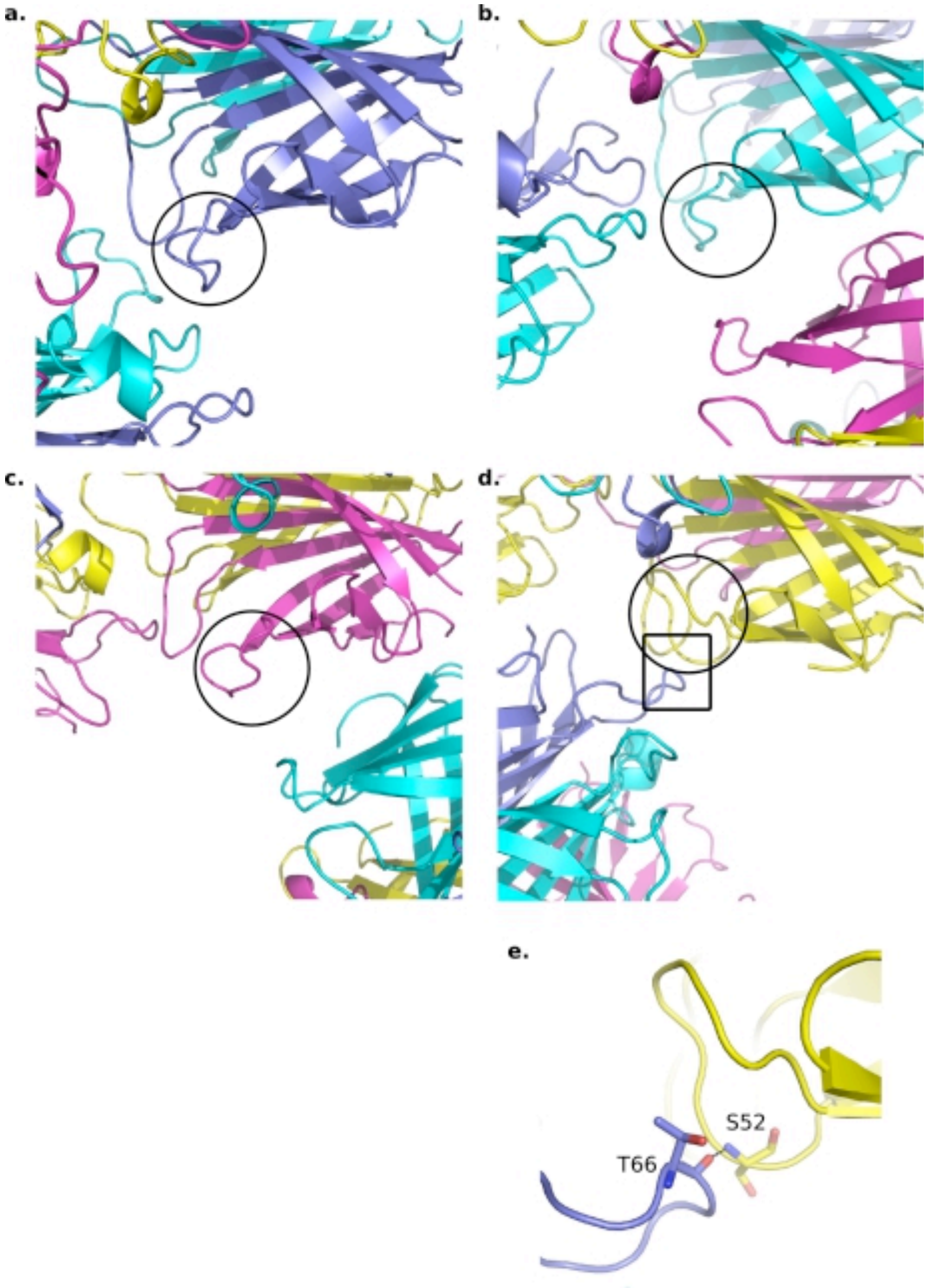

Supplementary Table 1. Root-mean-squared deviations (RMSD) (in Å) between four subunits (Chains A-D in this study) of monovalent streptavidin and the two previously reported streptavidin states: biotin-close conformation (Chain A of pdb id: 1MK5) or apo-open conformation (Chain D of pdb id: 3RY1). Streptavidin residues 16-130 are used for structural alignments.

	Close	Open
Chain A	2.46	0.72
Chain B	2.35	0.59
Chain C	2.35	0.63
Chain D	1.13	2.26
